# Species-specific optimization of oxylipin ionization in LC–MS: a design of experiments approach to improve sensitivity

**DOI:** 10.1007/s00216-025-05759-6

**Published:** 2025-02-01

**Authors:** Louis Schmidt, Ulrike Garscha

**Affiliations:** Department of Pharmaceutical/Medicinal Chemistry, Institute of Pharmacy, Greifswald University, 17489 Greifswald, Germany

**Keywords:** Design of experiments (DoE), Oxylipins, Lipidomics, UHPLC-ESI–MS/MS, Method optimization

## Abstract

Oxylipins are diverse bioactive signaling molecules, which occur in very low concentrations in complex matrices, posing challenges in achieving consistent and sensitive analysis. UHPLC-MS/MS is the preferred technique to separate and quantify these molecules, often optimized using a time-consuming trial-and-error approach. In this study, we applied the design of experiments (DoE) approach to systematically investigate the ionization properties of multiple oxylipin species. Fractional factorial and central composite designs were employed to detect relevant instrument parameters and optimize signal intensity in ESI–MS/MS analysis. Response surface modeling revealed distinct ionization and fragmentation behaviors between polar and apolar oxylipins, driven by their responses to interface temperature and collision-induced dissociation (CID) gas pressure. Particularly, prostaglandins and lipoxins benefit from higher CID gas pressure and lower temperatures compared to the lipophilic HODEs and HETEs to achieve optimal intensity in multiple reaction monitoring analysis. While global source parameters were optimized, analyte-specific entrance/exit potentials and collision energies required individual adjustments. The final method was applied to analyze seven oxylipin classes including leukotrienes, prostaglandins, lipoxins, resolvins, HETEs, HODE_S_, and HoTrEs. Although improvements in lower limits of quantification were modest (< 1 pg on-column), signal-to-noise ratios increased two-fold for lipoxins and resolvins and three- to four-fold for leukotrienes and HETEs, enhancing detection at trace levels. This DoE-guided strategy provides a powerful tool to improve UHPLC-MS/MS analysis of oxylipins across various instrument vendors, guiding the way towards inter-laboratory comparability.

**Graphical Abstract:**

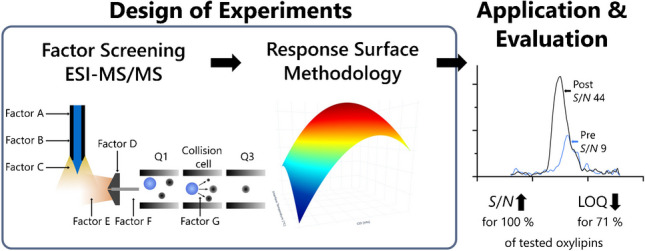

**Supplementary Information:**

The online version contains supplementary material available at 10.1007/s00216-025-05759-6.

## Introduction

In a wide range of organisms, from plants and fungi to mammals, a variety of molecules are released from cells to mediate transcellular signaling. A subset of these mediators are derivatives of polyunsaturated fatty acids (PUFAs), called oxylipins or lipid mediators. The best-known class of oxylipins are the eicosanoids, oxidized molecules of arachidonic acid (AA; 20:4; ω−6) [1]. Eicosanoids, including prostaglandins (PGs), leukotrienes (LTs), thromboxanes (TXs), and lipoxins (LXs), are involved in numerous physiological and pathophysiological processes, most notably in regulating immune response and maintaining homeostasis [1–3]. Other oxylipins are derived from eicosapentaenoic acid (EPA; 20:5; ω−3), docosahexaenoic acid (DHA; 22:6; ω−3), linoleic acid (LA; 18:2; ω−6), and linolenic acid (α-ALA; 18:3; ω−3 and γ-GLA; 18:3; ω−6); influence inflammation; and are discussed to be involved in the resolution phase [4]. Oxylipins engaged in the resolution of inflammation, also known as specialized pro-resolving mediators (SPMs), have gathered more attention due to their potential as diagnostic and prognostic biomarkers and therapeutics in chronic diseases [5–10]. These SPMs consist of lipoxins, maresins, protectins, and resolvins and occur in ranges of 0.5–100 pg/mL, depending on the matrix [11].

Besides enzymatic formation by lipoxygenases, cyclooxygenases, and cytochrome P450 (CYP) enzymes, all PUFAs can also undergo non-enzymatic oxidation. This process leads to the formation of more than 1000 structurally different oxylipins, which cover a broad spectrum of concentration levels and can have different effects on the human body, ranging from harmful to beneficial [12]. To date, the physiological effects and mediating receptors of many described oxylipins remain largely unknown. Advancing our understanding of these bioactive compounds requires the development and application of robust analytical methods for their precise identification and quantification.

To separate the oxylipins and isomers, (U)HPLC with C18 phases and triple quadrupole (QqQ) mass spectrometry (MS) in multiple reaction monitoring (MRM) mode is commonly used [13–18]. Due to improved separation efficiency and instrument sensitivity, even low abundant mediators can be detected and quantified. However, the amount of oxylipins detected in biological samples differs greatly when comparing the literature. These discrepancies, even between similar instruments, recently led to an investigation of published data, which revealed questionable data acquisition by some authors to quantify SPMs [19]. To address such issues, predefined quantification thresholds, such as signal-to-noise ratios (S/N), are essential for establishing a reliable limit of quantification (LOQ) [20]. To improve inter-laboratory comparability of analytical data, each laboratory should strive to use a sufficient LC method and adjust the MS parameters to achieve optimal ionization. Harmonization of sample preparation and LC method already improves data comparability, but when MS instruments from different vendors are used, more variance can be expected [21]. Therefore, each MS instrument should be optimized to obtain the most sensitive and robust data. In MS/MS analysis, this can be done in two steps. The first is to optimize the electrospray ionization (ESI) source to increase analyte ionization and to reduce ionization of impurities [22]. The second is to adjust the voltage potential within the instrument to focus the ions and direct them to the detector [23]. The second step is typically performed with vendor-specific modules implemented in the software. ESI optimization is often neglected, or if it is carried out, then in the form of a one-factor-at-a-time (OFAT) approach, optimizing one parameter after another, while the rest remains constant [24]. This procedure neglects potential interaction between instrument parameters, increases experiment numbers, consumes time, and in the end does not guarantee optimal results. A more systematic and statistically based methodology is the design of experiments (DoE) concept, where multivariate experiments are performed to fully evaluate the factorial parameters of the desired result [25].

In DoE, all parameters, called factors, are checked at predefined levels to investigate their impact on a specified response. Depending on the design, these screening designs allow a large number of factors to be screened with a few experiments that can be automated on an LC–MS instrument. Then, the factors that have a significant impact on the desired response are taken and optimized using an optimization design [25, 26]. From these experimental runs, the relation between the factors and the response can be described by a quadratic or polynomial equation. This is called response surface methodology (RSM) and a model is built where the optimal level of each parameter can be calculated [27]. Some studies have applied DoE principles to optimize ESI; however, these often fail to demonstrate a significant overall improvement in ionization efficiency compared to baseline methods [28–32].

In the present work, we aim to enhance the sensitivity of our LC–MS/MS method by improving analyte ionization and fragmentation. For this purpose, we employ the DoE approach to understand the ionization behavior of different oxylipin species and to develop an RSM to calculate optimal instrument settings. Finally, we compare the new optimized method to baseline settings and evaluate sensitivity improvements through calibration curves.

## Material and methods

### Materials

Acetonitrile (ACN), methanol (MeOH), and acetic acid, all in LC–MS grade, were purchased from Fisher Scientific (Schwerte, Germany). H_2_O LC–MS grade was from VWR (Darmstadt, Germany). The oxylipin standards were all from Cayman Chemicals and distributed by Biomol (Hamburg, Germany).

### LC–MS/MS analysis

All measurements were conducted on a Nexera LC-40 UHPLC from Shimadzu (Kyoto, Japan). The system comprised two LC-40B X3 delivery modules, a SIL-40C X3 autosampler, a CT-40S column oven, and an SPD-40 UV–Vis detector, an FCV-20AH2 valve connected to a LCMS-8060 QqQ mass spectrometer with an ESI source. Chromatographic separation was achieved using a Waters ACQUITY UPLC BEH C18 column (2.1 × 100 mm, 1.7 µm) equipped with a Waters ACQUITY in-line filter (0.2 µm). Solvents, flow rate, and gradient followed the procedure described by [13]. Solutions A and B were water and ACN/MeOH (80/15), each supplemented with 0.1% acetic acid. The oven temperature was set to 40 °C and a flow rate of 0.3 mL/min was applied. The gradient elution started with 25% B and remained constant until 1 min. From 1 to 1.5 min, the concentration of B was increased to 30%. Between 1.5 and 10 min, B was further increased to 53%. The gradient continued with an increase to 68% B within additional 9.5 min, followed by a rise to 95% B from 19.5 to 24.5 min. This 95% B concentration was maintained for 2.5 min before B was set again to 25% for re-equilibration.

Oxylipins were detected in MRM with argon as collision gas. Dwell times were optimized to achieve 15–20 data points per peak, and a detection window of ± 30 s around the expected retention time was set. For each analyte, three transitions were monitored: two qualifier ions and one quantifier ion. Ion transitions, voltage potentials, and collision energies (CEs) were optimized for each analyte using the instrument’s optimizer tool and were cross-referenced to literature data. Detailed retention time, quantifier transition, voltage potentials and CEs are summarized in Table S1. Peak integration, calibration curve, *S*/*N*, and accuracy calculation were performed in LabSolution (version 5.97).

### Screening design

**Fig. 1:**
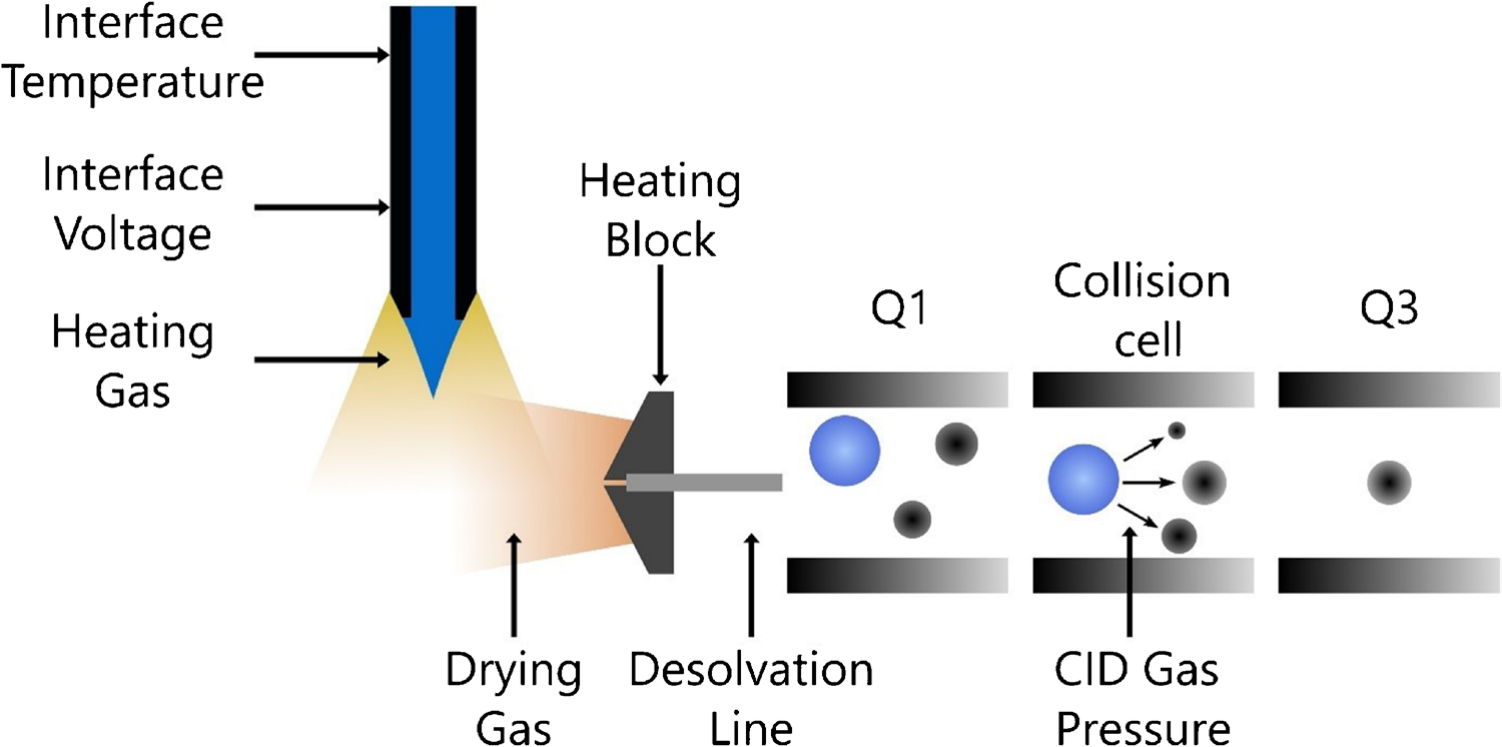
Electrospray ionization source and QqQ of the LCMS-8060. Tested and optimized instrument parameters are marked with arrows

A fractional factorial design (FFD) with resolution IV was employed to screen the most relevant factors contributing to signal intensity. Design matrix and data analysis were performed using MODDE Pro version 13 (Sartorius, Göttingen, Germany). Since ion intensity in MS/MS analyses depends on both the ionization process in the ion source and the fragmentation in the collision cell, both sections of the instrument were evaluated. A schematic setup of the ESI and QqQ, detailing the factors investigated in this study, is presented in Fig. 1. Nebulizing gas (NG) flow was maintained at 3 L/min for all experiments.

Following the initial determination of factor levels, each factor was tested at two levels (− 1, 1), and three center points (0) were included to assess system robustness. The factors and their corresponding levels are summarized in Table 1. For the experiments, a mixture containing five oxylipin species, prostaglandin E_2_-d_4_ (PGE_2_-d_4_), lipoxin A_4_-d_5_ (LXA_4_-d_5_), leukotriene B_4_-d_4_ (LTB_4_-d_4_), 9-hydroxyoctadecadienoic acid-d_4_ (9-HODE-d_4_), and 5-hydroxyperoxyeicosatetraenoic acid-d_8_ (5-HETE-d_8_), in MeOH at a concentration of 7.5 ng/mL was used, with 5 µL injected per run for both screening and optimization. Each experimental run was conducted in duplicate, and the mean peak height was used for calculations.

**Table 1:** Instrument settings and levels tested in screening design

Parameter	Abbreviation	− 1	0	1
Interface voltage (kV)	IntV	2	3	4
Interface temperature (°C)	IntT	200	300	400
Heating gas (L/min)	HG	5	7.5	10
Drying gas (L/min)	DG	5	7.5	10
Heating block (°C)	HB	300	400	500
Desolvation line (°C)	DL	200	250	300
CID gas pressure (kPa)	CID	140	205	270

### Optimization

Following the identification of critical factors, interface temperature (IntT), interface voltage (IntV), and collision-induced dissociation (CID) gas pressure were optimized using a central composite design (CCD). This design assesses each factor at three levels (− 1, 0, 1) with two additional star points (− α, + α**)** beyond the cubic space to gather more comprehensive data on the system. A distance of 1.35 was used for the star points, and three center points at level 0 were included to assess system robustness. The selected levels are summarized in Table 2. Note, due to instrument limitation, IntT could not be higher than 400 °C, and therefore the + α level could not be investigated. Design matrix, RSM, and Monte Carlo simulations were performed using MODDE. The settings for heating block (HB), desolvation line (DL), heating gas (HG), and drying gas (DG) were 400 °C, 250 °C, 10 L/min, and 10 L/min, respectively. The optimized parameter values were determined to be 222 kPa for CID gas pressure, 380 °C for IntT, and 2 kV for IntV.

**Table 2:** Factor levels tested in CCO design

Parameter	Abbreviation	− α	− 1	0	1	+ α
CID gas pressure (kPa)	CID	120	140	195	250	270
Interface temperature (°C)	IntT	282	300	350	400	-
Interface voltage (kV)	IntV	1.8	2	2.5	3	3.2

Afterwards, RSM was employed to evaluate the interaction between CE and CID gas pressure and determine whether further adjustments were necessary. A multilevel D-optimal design was applied with CE ranging from 12 to 28 eV and CID varying from 190 to 250 kPa. The CE range was defined as the minimum and maximum values across all oxylipins (± 3 eV), determined with the automatic optimization software tool prior to this study. To compare the RSM results with a traditional OFAT approach, the same sample was analyzed at a fixed CID pressure of 222 kPa, with CE incrementally varied in 2 eV steps, each step measured in duplicate.

### Application and sensitivity evaluation

After optimizing the instrument parameters, the impact of these adjustments was evaluated using two different approaches. First, 5 µL of the same oxylipin mix used in the DoE was analyzed under standard MS conditions, with each factor individually set to its optimal value, as well as under fully optimized conditions. Detailed instrument settings are summarized in Table S4. Each setup was tested in triplicate, and the mean peak height and mean AUC acquired under standard conditions were set as baseline signals. To assess whether the optimization process improved sensitivity, a mixture of several oxylipins, including 20-hydroxyleukotriene B_4_ (20-OH-LTB_4_), prostaglandin E_2_ (PGE_2_), prostaglandin D_2_ (PGD_2_), thromboxane B_2_ (TXB_2_), prostaglandin F_2α_ (PGF_2α_), lipoxin A_4_ (LXA_4_), resolvin D_5_ (RvD_5_), 6-*trans*-leukotriene B_4_ (*t*-LTB_4_), leukotriene B_4_ (LTB_4_), 9-hydroxyoctadecadienoic acid (9(*S*)-HODE), 13(*S*)-hydroxyoctadecatrienoic acid (13(*S*)-HoTrE), 15(S)-hydroxyeicosatetraenoic acid (15(*S*)-HETE), 12(*S*)-hydroxyeicosatetraenoic acid (12(*S*)-HETE), and 5(*S*) − hydroxyeicosatetraenoic acid (5(*S*)-HETE), was sequentially diluted in MeOH, ranging from 3.125 ng/mL to 6.1 pg/mL. Ten microliters of each level was injected and measured with the standard method (pre-optimization) and the final method (post-optimization). Each calibration point was measured twice in alternating sequence to minimize time-dependent instrument shifts. Calibration curves were calculated with linear least square regression (weighting: 1/x) and the LOQ was determined by a *S*/*N* ≥ 5 and a back-calculated accuracy of ± 20% as recommended by the European Medicines Agency [20].

## Results and discussion

### Ion source and preliminary screening

The objective of this study was to statistically and graphically assess the influence of instrumental parameters on the ionization of various oxylipin species in LC–MS/MS analysis to enhance sensitivity. To investigate ionization and fragmentation behavior, a closer examination of the adjustable instrument parameters related to the ESI source and QqQ was necessary. The ESI source of the LCMS-8060 features an orthogonal design, in which the spray enters the MS at a 90° angle (Fig. 1). This approach directs only the edge of the spray cone into the vacuum region, enabling higher LC flow rates and reducing instrument contamination.

In the LCMS-8060 ESI source, the sample is introduced through a heated capillary (regulated by IntT) and enters the ionization chamber upon a high voltage (IntV) is applied. HG and DG (both nitrogen) along with a HB surrounding the MS inlet facilitate solvent evaporation. Single analyte ions and small solvent clusters pass through a heated DL before entering the QqQ. In Q1, precursor ions are selected, then fragmented in Q2 (collision cell) using analyte-specific CE and collision-induced dissociation (CID) with an inert gas. In Q3, the specific fragment ions are filtered and further analyzed. Therefore, optimizing ionization and fragmentation conditions is critical for maximizing the signal intensity of the fragment ions.

In the preliminary screening, the factors and ranges of all instrument parameters affecting signal intensity in Q3 were examined to set the limits in the DoE experiment. Seven of the eight factors (Table 1) were selected for further examination, while NG, which surrounds the ESI capillary, was excluded from optimization as it was already set to its optimal condition. NG is crucial for stabilizing the electrospray at higher flow rates and enhancing solvent dispersion, facilitating the formation of a fine mist of charged droplets. These droplets can, depending on the mechanism, either release a single ion from the surface into the gas phase through electrostatic repulsion (ion evaporation mechanism), or undergo consecutive fissions until a charged droplet containing one analyte molecule remains (charge residue model) [22, 33]. Although NG values typically range from 1 to 3 L/min, a notable decrease in signal intensity was observed when the flow was set below the standard 3 L/min. Consequently, NG was kept at 3 L/min for all experiments.

To determine whether changes in CID pressure affect fragment ion specificity, product ion scans of several analytes were performed at various pressure settings (140–370 kPa) (data not shown). No changes were detected in the specific, structure-related fragments. However, the intensity of the fragments varied with pressure. Higher CID gas pressures promoted the formation of smaller fragments through successive fragmentation events. Above 270 kPa, the total intensities of all fragment ions decreased. Therefore, 270 kPa was set as the upper limit for screening.

### Fractional factorial screening design

A FFD with resolution IV was chosen to assess the importance of each factor, while distinguishing main effects from two-factor interactions. Given the complex nature of ESI, factor interactions can mask individual effects. For instance, increasing the IntT while decreasing HG could lead to unchanged solvent evaporation, masking the potential benefit of IntT on gas-phase ion formation. Reduced screening designs, such as D-optimal or Plackett–Burman, decrease the number of experiments and safe time, but also increase the confounding of main effects with two-factor interactions and therefore could lead to inaccurate conclusions [34]. A FFD on the other hand allows the evaluation of main effects and two-factor interactions, even though these interactions may still be partially confounded. Although this design increased the number of experiments, the screening was completed within 24 h with 19 runs conducted in duplicate and each lasting 30 min. Examination of the center points showed excellent reproducibility over the tested range with minimal deviations (data not shown).

The complete design matrix and factor values are detailed in Table S2. Analysis of variance (ANOVA) was used to determine significant main effects on ion intensity in Q3. Figure S1a shows the coefficient plots of the raw data, revealing that, depending on the analyte, only IntT and/or CID have a positive influence on the signal intensity. Error bars crossing the *X*-axis indicate factors without significant effects, allowing their removal to reduce model complexity (principle of parsimony [35]) and to improve model correlation (*R*^2^) and predictability (*Q*^2^). For four analytes, the main factors IntT and CID were significant, while for 5-HETE-d_8_ only IntT was significant as a main factor.

Additional model refinement (Figure S1b) was achieved by including quadratic and interaction terms. However, due to the limitations of the FFD, quadratic terms exhibited large errors and therefore results should be interpreted with caution. The inclusion of interaction terms improved the model fit to some extent, although, in an FFD with resolution IV, each interaction is inherently confounded with two others. For 5-HETE-d_8_, an interaction of IntT × IntV was observed, which is confounded with CID × HB and DL × HG. Similarly, for LTB_4_-d_4_ and 9-HODE-d_4_, an interaction between CID × IntT was found, confounded with IntV × HB and HG × DG. It was considered to analyze each interaction individually but this was not feasible due to the small effect size of these interactions and the substantial time investment required. Based on the results from the 19 screening runs, three factors—IntT, CID, and IntV—were selected for further optimization, as lower interface voltages combined with higher IntT could provide beneficial effects.

### Optimization, RSM, and fragmentation behavior

To determine optimal settings, the three remaining factors were further examined using a CCD. Compared to other RSM designs, such as Box-Behnken (BBD), CCD enables the exploration of factors at combined minimum and maximum levels [26, 36]. Figure S2 illustrates the design region, with the blue spheres representing hypercube-forming experiments and yellow spheres indicating the additional axial points to increase robustness, flexibility, and rotatability. In the initial screening, high IntT combined with low IntV hinted a beneficial interaction, justifying further investigation through CCD. The design matrix consists of 17 runs (Table S3) and was analyzed in 17 h with each experiment done in duplicate.

**Fig. 2:**
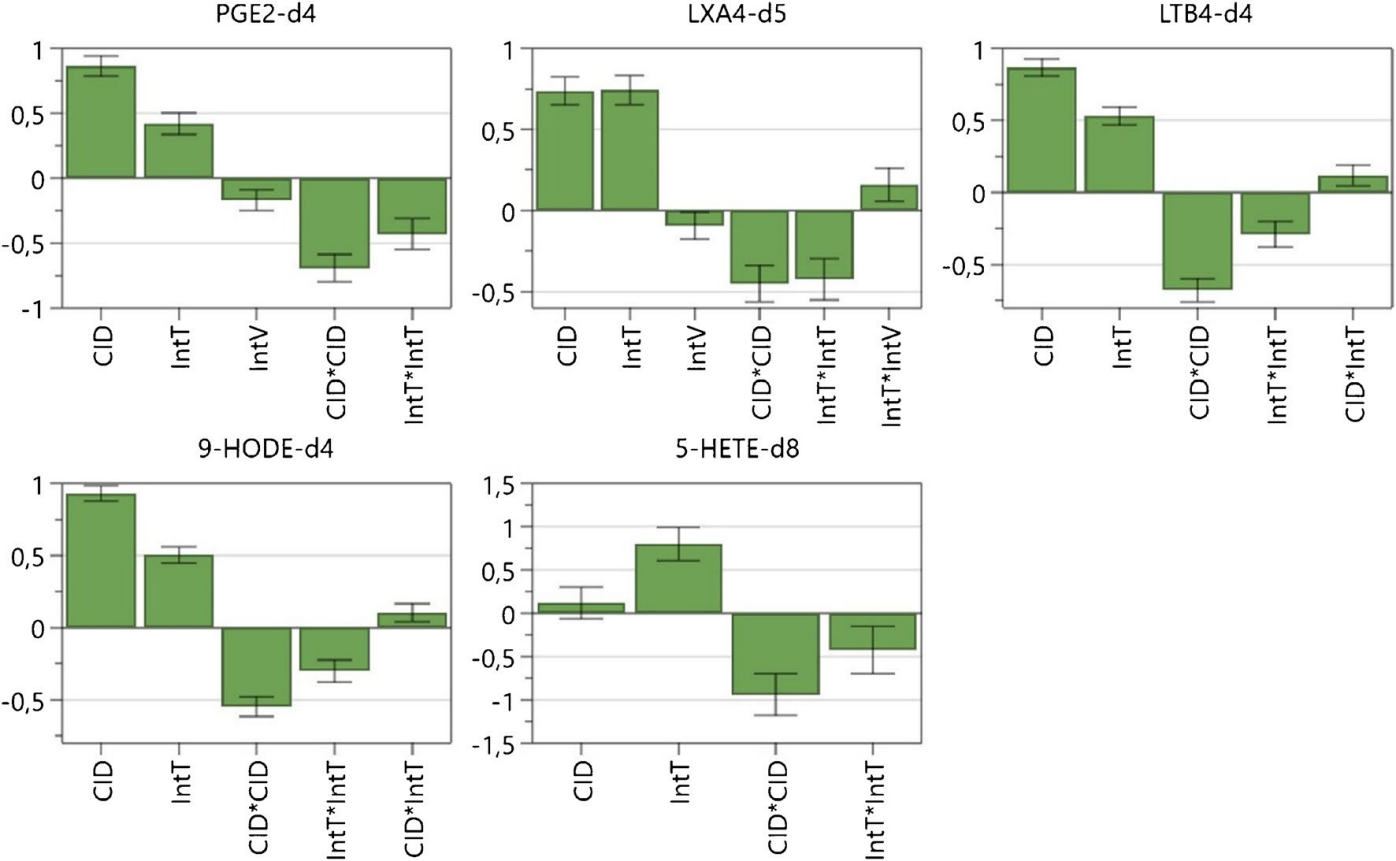
Coefficient plots for the investigated factors in CCD. Abbreviations are explained in Table 1. The error bars represent the 95% confidence interval

The collected data was analyzed using ANOVA, and non-significant terms were removed to improve model accuracy. All resulting models demonstrated high *R*^2^ (> 0.94), *Q*^2^ (> 0.86), and reproducibility (> 0.97), and a non-significant lack of fit (*p* > 0.05). Coefficient plots (Fig. 2) provided valuable insights into the ionization and fragmentation behavior of various oxylipin classes. As expected, main factors significantly influenced peak height to a large extent. Higher CID and IntT values enhanced Q3 signal intensity for four out of five analytes, visible at the positive coefficient values. The negative coefficient value of IntV indicates that higher voltages decrease signal intensity.

For CID and IntT, the quadratic terms (CID × CID and IntT × IntT) were significant, indicating a curved response with an optimal point between the minimum and maximum levels tested. Higher IntT enhances the desolvation of the mobile phase, yielding more gas-phase ions that can enter the MS. While increased IntT generally enhances signal intensities, it can also induce thermal stress and in-source fragmentation in some analytes, reducing signal intensity, which will be discussed later [37–40]. Hence, careful optimization should be carried out.

IntV was included in the CCD due to a slight improvement in the FFD screening but it was expected to have a minor influence compared to the other two factors. Indeed, a lower voltage slightly enhanced the ionization of polar oxylipins like PGE_2_-d_4_ and LXA_4_-d_5_, visible by negative coefficient values (increased voltage reduced the peak height). The interaction of IntT × IntV was only significant for LXA_4_-d_5_ with a positive effect value, meaning a slight benefit at higher temperatures with higher voltage was examined. This was only the case for IntT 400 °C; below this point lower voltage was superior.

Interestingly, only for LTB_4_-d_4_ and 9-HODE-d_4_, the IntT × CID interaction was significant. A higher IntT supported peak height by increasing gas phase ion production, while elevated CID enhances analyte cleavage into specific fragments. Although this suggests a potential positive interaction between high IntT and CID across all oxylipins, data show these processes occur independently.

**Fig. 3:**
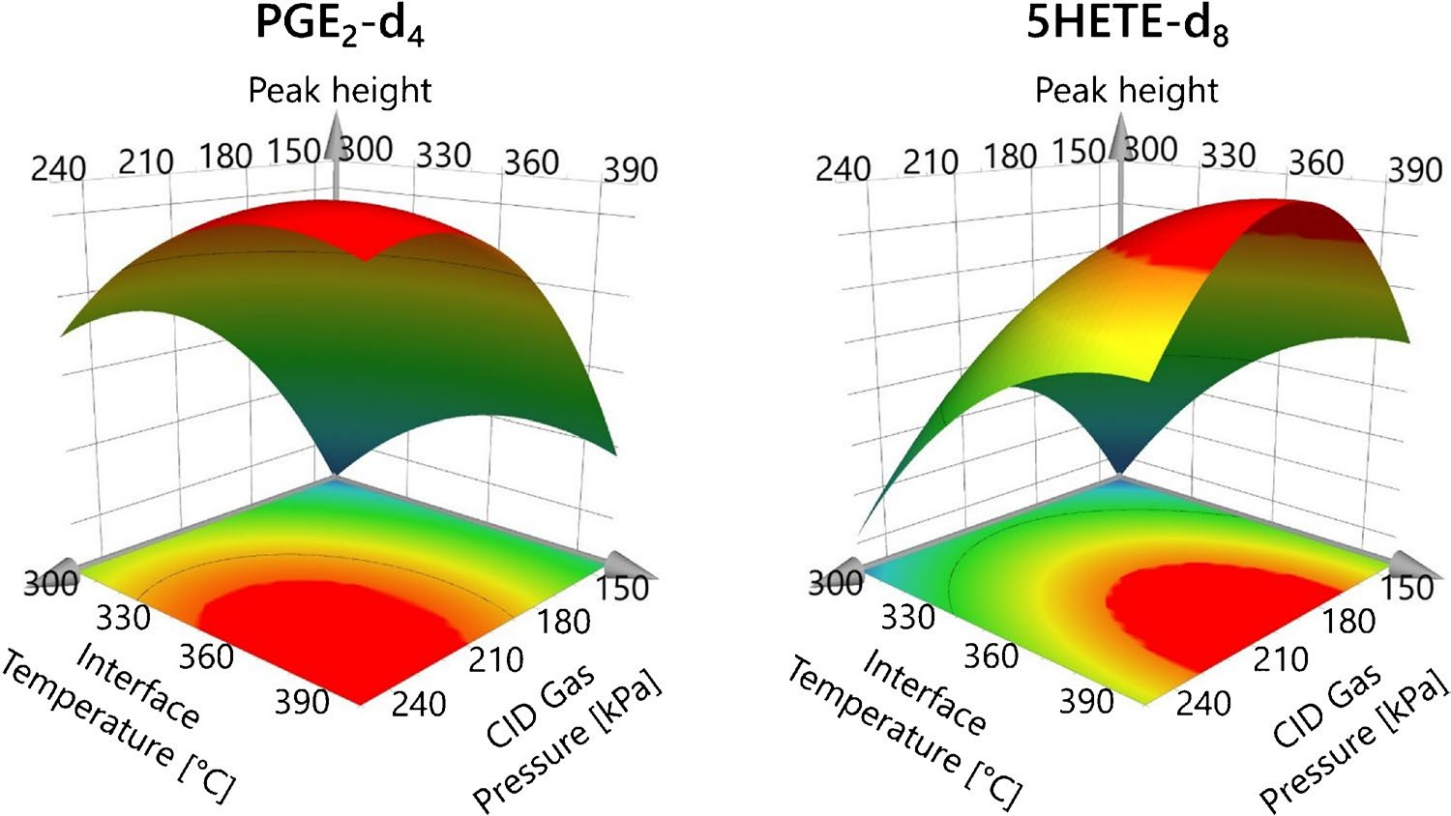
Response surface plots for PGE_2_-d_4_ (left) and 5-HETE-d_8_ (right) obtained from the CCD optimization runs at 2 kV IntV. The red area indicates the region of optimal ionization and fragmentation. Ion intensity in Q3 of PGE_2_-d_4_ specific fragments benefits from lower temperature and higher CID gas pressure, while the ion intensity for 5-HETE-d_8_ increases with higher IntT and lower CID gas pressure

After model refinement, response surface plots were generated to visualize the optimal conditions. Figure 3 compares response surface plots at a fixed IntT of 2 kV for the hydrophilic PGE_2_-d_4_ and the lipophilic 5-HETE-d_8_, because these were the first and last eluting analytes and showed the least similarity in structure. For PGE_2_-d_4_, the curvature of the plot indicated an optimum at a CID between 210 and 270 kPa and an IntT between 360 and 390 °C. In comparison, 5-HETE-d_8_ tolerated higher temperatures, visible at a plateau over 390 °C, but lower CID between 180 and 210 kPa. Due to instrument limitation, a higher IntT was not evaluated, so the optimal temperature may exceed the tested range.

These differences may arise from distinct fragmentation mechanisms. Compounds containing cyclopentanone structures, such as prostaglandins, isoprostanes, and jasmonic acid precursors often disintegrate through neutral loss resulting in unspecific fragments (e.g., [M-H–H₂O]⁻ and [M-H–H₂O-CO₂]⁻), a process observed in both the collision cell and in-source fragmentation [13, 14, 23, 41–43]. These in-source fragmentation can occur, when the internal energy (the total energy of a species above its electronic, vibrational, and rotational ground state [44]) of a molecule increases beyond a stability threshold, resulting in the cleavage of unstable bonds. A higher IntT increases the thermal and kinetic energy of the analyte. This leads to increased vibrational excitation and, in combination with subsequent collision with neutral gas molecules, promotes in-source fragmentation. Increasing the ionization voltage and the voltage differences within the lens system enhances ion velocity thereby raising internal energy [23, 44–46]. The influence of temperature and voltage is more important for labile molecules, which can decompose quickly and release neutral molecules like CO_2_ and H_2_O. The cyclopentanone structure could lead to a less thermal-stable molecular ion, which decomposes more likely than linear structured molecules. This instability was evident in the main effects plot (Figure S3), where linear structures such as LXA_4_-d_5_, LTB_4_-d_4_, 9-HODE-d_4_, and 5-HETE-d_8_ were more robust at higher temperatures, while only PGE_2_-d_4_ exhibited decreased response at elevated IntT. Sajiki and Kakimi utilized this in-source fragmentation to identify PGE_2_ in algae with increasing the cone voltage to induce the loss of H_2_O and CO_2_ before the molecule enters the MS [47]. In contrast to the broader literature, only a limited number of publications document IntT and IntV settings specifically for the Shimadzu QqQ system. When given, the IntT was reduced to 275 °C or 150 °C and IntV was increased to 4 kV, compared to the standard settings of 300 °C and 3 kV [48, 49]. Tsuji et al. used IntT at 300 °C, but decreased IntV to 2 kV, probably to the reduced flow rate of 50 µL/min [50]. This highlights the cross play between IntT and IntV. Increasing both values leads to a decrease in overall signal intensity.

Regarding the optimized CID setting, PGE_2_-d_4_ tolerated higher gas pressure to form the specific fragments, while 5-HETE-d_8_ benefits from slightly lower CID values. Main effect plots (Figure S4) show the optimal CID shifting with analyte polarity: PGE_2_-d_4_ peaks at 230 kPa, while 5-HETE-d_8_’s optimum is around 200 kPa. This could be due to the specific MRM transition used in this experiment. 5-HETE-d_8_ dissociates due to an α-cleavage adjacent to the hydroxy function, while PGE_2_-d_4_ forms the unspecific [M-H-H_2_O-CO_2_]^−^ fragment. The cyclopentanone moiety may exhibit greater resilience to higher CID energies before dissociating into smaller, less specific fragments. The small hydroxy-carboxylic acid fragment from 5-HETE-d_8_ can quickly decompose under higher CID into CO_2_, H_2_O, and hydrocarbon, resulting in a loss of sensitivity. These different behaviors of oxylipin classes were partially already investigated in the literature. Kutzner et al. optimized the ionization of SPMs with flow-injection analysis on a QTRAP 6500 (Sciex). They also noticed that the trihydroxylated RvD_2_ tolerated higher CID conditions compared to the dihydroxylated RvD_5_ and RvE_1_ [24]. In our study, increasing CID from 140 to 250 kPa at an IntT of 400 °C improved peak height by 51%, while raising IntT from 300 to 400 °C at a CID of 250 kPa resulted in a 32% improvement on average. CID pressure, therefore, emerged as the most critical parameter for optimizing MS/MS intensity, assuming constant CE and Q1/Q3 potentials.

### Design space and CE evaluation

**Fig. 4:**
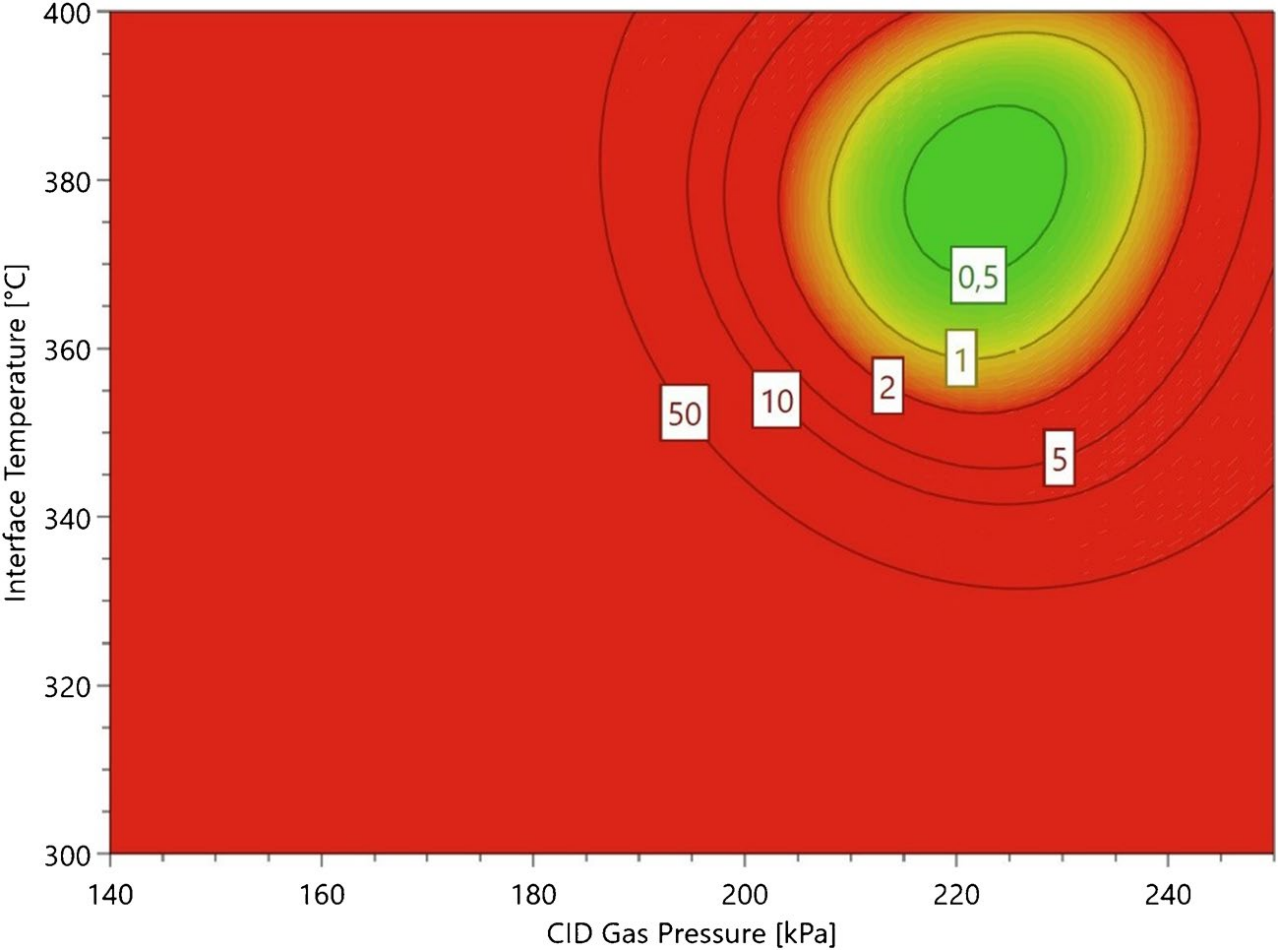
Design space obtained by Monte Carlo simulation. The green area shows the region, where the probability of failure is < 1%. Failure is defined as the deviation of a defined analyte response. To achieve the optimal intensity of all oxylipins included in the DoE, the instrument parameter for CID should be around 220 kPa, while the IntT should be 380 °C

One of the key advantages of the DoE approach is the creation of a design space, which defines the multidimensional combination of selected factors that achieve the desired response with an acceptable level of risk for failure [51, 52]. To determine optimal parameter settings, a target peak height (calculated from RSM) was selected and weighted for each analyte, based on its significance. We prioritized optimal ionization and fragment conditions for prostaglandins and lipoxins over HETEs and HODEs given their lower abundance [43, 53–57]. The design space, shown in Fig. 4, was generated through Monte Carlo simulation, revealing optimal conditions at a CID of 222 kPa, an IntT of 380 °C, and an IntV of 2 kV, with a probability of failure below 1%. These results were partially in agreement with literature data, where other authors also used CID values on the Shimadzu LC/MS instrument at around 230 kPa for oxylipin analysis [48, 58]. Contrary, our study highlights the advantages of higher IntT and lower IntV, diverging from other studies that typically employed lower IntT and higher IntV settings [48, 49].

To determine whether the reduction in CID from 270 to 222 kPa leads to a necessary adjustment of CE values, RSM was employed to develop a suitable model for each of the five oxylipins. A D-optimal design was selected, involving 14 runs, each performed in duplicate, leading to a total analysis time of 14 h. The response contour plots corresponding to these models are shown in Figure S5a, with the red areas highlighting regions of maximum product ion signal intensity. After model refinement, CE emerged as a significant factor for all five analytes. For LXA_4_-d_5_ and 5-HETE-d_8_, CID also showed an impact and slightly improved the model, although its influence was less pronounced compared to the factorial analysis. However, in this experiment, CID had minor to no effect, visible in contour plots, where the optimal region was solely dependent on CE. This suggests that the dominant effect of CE may overshadow any potential contribution from CID. While the response surface models predicted an ideal CE region, the range of CE values was too broad to determine an optimal setting. To refine the CE determination and validate the RSM results, each analyte was analyzed at a fixed CID pressure of 222 kPa, with CE values varying from 12 to 28 eV. Figure S5b presents the data, normalized to the highest signal at 100%. For all analytes, the CE values that led to optimal fragmentation were within the predicted range of the contour plots. However, even small deviations of 2 eV from the optimum resulted in a reduction of 7–15% in peak height. Notably, the optimal CE values for four of five analytes aligned closely (only 2 eV deviation for LXA_4_-d5) with the values determined at a CID pressure of 270 kPa, indicating that adjustments to CE are mostly independent of CID pressure. These findings align with results from a previous study [24], where CE optima may vary within a narrow range of approximately 2 eV depending on the CID gas setting. Based on this experiment, we do not recommend optimizing CID and CE together, as the dominant influence of CE tends to overshadow the effect if CID. In addition, the individual reaction of analytes to small changes in CE requires careful adjustments, which should be carried out in small steps.

### Application and sensitivity evaluation

**Fig. 5:**
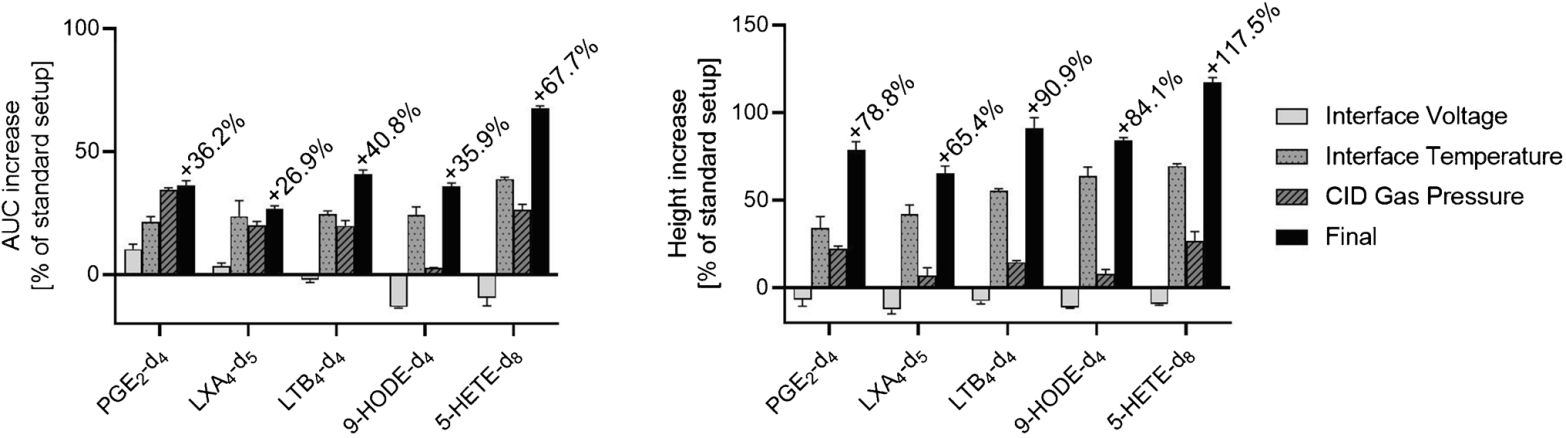
Comparison of peak height and AUC of five deuterated standards at standard conditions (3 kV, 300 °C, 270 kPa), optimized conditions (2 kV, 380 °C, 222 kPa), and each parameter at their individual optimum while the rest remained at standard level. Peak height and AUC of the standard setting were set as baseline. The data show the improvement of *n* = 3 measurements with mean ± SD. Notably, the peak height improved at 65–117%, while AUC demonstrated less improvement with 26–67%

To assess whether the optimized instrument settings provided a significant improvement in analyte response, we conducted a sensitivity evaluation comparing the optimized conditions (2 kV, 380 °C, 222 kPa) to standard conditions (3 kV, 300 °C, 270 kPa). Initially, the sample mixture used for DoE was analyzed under both standard and optimized conditions, as well as at the optimal level for each parameter while keeping the other parameters at their standard levels. Data obtained under standard conditions was set as the baseline. Interestingly, both peak height and AUC improved for all analytes though not to the same extent (Fig. 5). AUC, which is directly correlated with the amount of analyte molecules, improved by 26.9–67.7%, while peak height increased by 65.4 to 117.5%. Notably, IntV alone decreased peak height for all analytes but improved AUC for polar oxylipins. This phenomenon may arise from enhanced ionization of the molecules, combined with increased contamination from coeluting compounds that raise the baseline. In contrast, apolar oxylipins did not benefit from lower IntV, either in peak height or AUC. For these compounds, ionization was insufficient unless IntT was also increased simultaneously.

The most significant gains in sensitivity were attributed to IntT. As previously discussed, a higher IntT promotes better desolvation, which enhances ion generation in the gas phase while reducing the number of solvent molecules entering the MS, thereby lowering background noise. CID was also a crucial parameter, increasing AUC for all analytes except 9-HODE-d_4_, although it only slightly improved peak height. Since CID primarily influences the specific fragmentation, it is directly correlated with the amount of product ions in Q3 and therefore with the AUC, but it has no impact on the ionization process. Consequently, solvent and impurity molecules that entered the MS were not sufficiently reduced contributing to baseline noise that diminished the overall increase in peak height. Nevertheless, nearly all analytes exhibited over-additive effects of combined parameters changes on peak height, which resulted from an improved ionization, reduced ion source contamination, and enhanced specific fragmentation. This leads to more specific ions entering the Q3. Additionally, better mobile phase evaporation led to reduced noise levels and improved signal-to-noise (*S*/*N*) ratios.

As a final assessment, the standard and optimized methods were compared using calibration curves, for 14 oxylipins ranging from polar to apolar metabolites. The calibration curves were measured four times, twice under standard and twice under optimized settings in alternating order to reduce time-dependent instrument shifts. Table 3 shows that 10 out of 14 analytes exhibited improved LOQ values, often by a factor of 2, and all 14 showed enhanced *S*/*N* ratios at their pre-optimization LOQ levels. Comparison of pre- and post-optimization MS chromatograms for each analyte are included in the Supporting Information (Figure S6). Among all analytes, LTB_4_, *t*-LTB_4_, and the included HETEs exhibited the most significant benefits from optimization with *S*/*N* ratios up to five times higher compared to standard settings. This is particularly important for low abundant analytes like leukotrienes, lipoxin, resolvins, and other SPMs. In 2023, some authors demanded critical evaluation of raw LC/MS data with predefined thresholds for LOQ, to reduce data manipulation and support interlaboratory comparison [19]. Often *S*/*N* ratios were used to determine LOQ levels (Table 2 in [11]), but even with the same instrument, some deviation occur. Enhancing *S*/*N* ratios, particularly at low concentrations, is crucial for minimizing false-negative results, improving the robustness of data acquisition and reducing inter-laboratory deviation. The applied DoE approach demonstrated that oxylipin ionization may differ, but graphical and statistical tools can be applied to select the instrument parameters that guarantee a robust data acquisition.

**Table 3:** LOQ and *S*/*N* comparison of calibration curves of 14 oxylipins acquired under standard and optimized UHPLC-ESI–MS conditions. For all measurements, CE and Q1 and Q3 voltages remained the same. Ten analytes showed improved LOQ values, while all 14 improved *S*/*N* ratios at a defined concentration. LOQ was defined as the lowest concentration where *S*/*N* > 5 and the back calculated accuracy of 80–120%

Analyte	Limit of quantification (pg on column)		*S/N*^a^	
	Pre-optimization	Post-optimization	Pre-optimization	Post-optimization
20-OH-LTB_4_	0.5	0.5	5.9	17.2
TXB_2_	1	0.5	5.2	12.5
PGF_2α_	2	1	8.1	11.7
PGE_2_	1	0.5	8.9	12.4
PGD_2_	1	1	12.8	15.7
LXA_4_	1	1	5.7	10.1
RvD_5_	1	0.5	5.2	11.5
*t*-LTB_4_	1	0.5	10.8	30.2
LTB_4_	1	0.5	7.1	24.7
13-HoTrE γ	4	2	5.8	14.6
9-HODE	1	1	15.6	32.7
15-HETE	2	1	6.9	25.9
12-HETE	2	0.5	8.9	36.7
5-HETE	1	0.24	8.9	44.2

^a^*S*/*N* ratio at the pre-optimization LOQ

## Conclusion

In this study, we present a statistically and graphically based approach, using design of experiments (DoE) principles, to investigate oxylipin ionization behavior aiming to ensure robust and sensitive quantification within our laboratory. Traditional methods, such as flow injection analysis and the one-factor-at-a-time (OFAT) approach, often require numerous individual runs for MS setting optimization, failing to replicate the actual conditions under which samples are analyzed. These methods fail to account for solvent composition and column effects, potentially resulting in misleading conclusions. In contrast, our approach utilized the same column and mobile phase conditions as those used for biological samples, thereby significantly reducing the risk of random error. Moreover, we utilized Q3 ion intensity after MRM to assess factor impact, as this metric encompasses both ion formation in the electrospray ionization (ESI) source and fragmentation efficiency in the collision cell. By employing a DoE approach, we were able to analyze multiple analytes simultaneously, leveraging chromatographic separation for systematic evaluation and detection of structure-specific ionization behaviors. The new method significantly enhances the sensitivity and robustness of oxylipin quantification, providing a solid foundation for future studies on the complex behaviors of oxylipins and their derivatives.

## Supplementary Information

Below is the link to the electronic supplementary material.

Supplementary file1 (DOCX 1400 KB)
