# Supplementary material for: Species-specific optimization of oxylipin ionization in LC–MS: a design of experiments approach to improve sensitivity
Source: Anal Bioanal Chem. 2025 Feb 1;417(9):1807–18. doi: 10.1007/s00216-025-05759-6 (PMC11913994; doi:10.1007/s00216-025-05759-6)
Supplement: Supplementary file 1 — Supplementary file1 (DOCX 1400 KB) [file 216_2025_5759_MOESM1_ESM.docx]

**Species-specific optimization of oxylipin ionization in LC-MS: A design of experiments approach to improve sensitivity**

Louis Schmidt ^1^, Ulrike Garscha ^1,*^

^1^Department of Pharmaceutical/Medicinal Chemistry, Institute of Pharmacy, Greifswald University, 17489 Greifswald, Germany

^*^Correspondence: ulrike.garscha@uni-greifswald.de


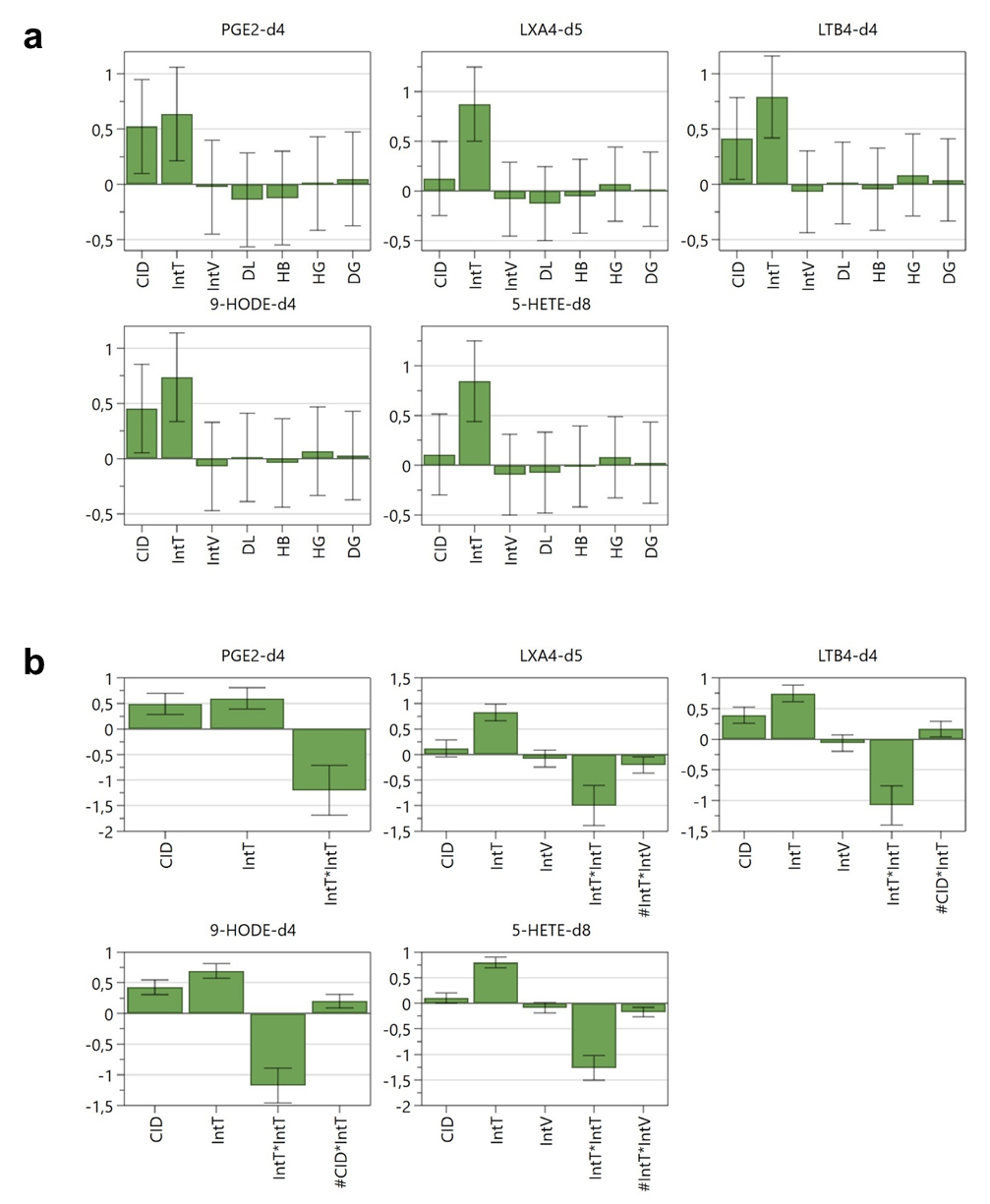


**Figure S1** Coefficient plots (normalized and scaled) for the factors contributing to enhanced ionization in the fractional factorial screening design (**a** without data processing, **b** after model fit).


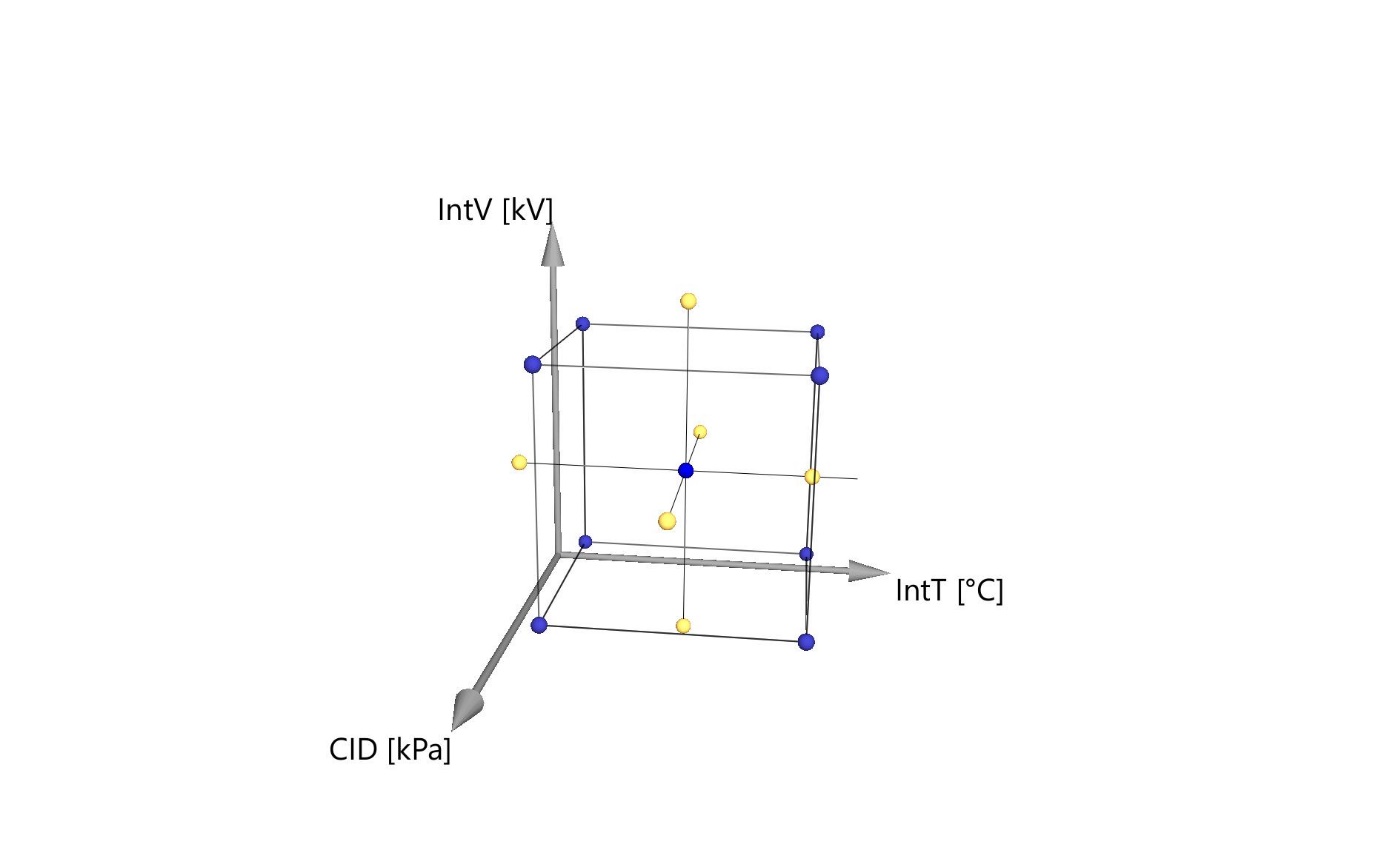


**Figure S2** Design Region for the optimization. The blue spheres highlight the center point and the edges of the cube. Six additional runs with α=1.35 outside the hypercube were done, shown in yellow. Note, due to instrument limitation the IntT value of +1.35 was not tested and the maximum value was set to instrument limit of 400°C.


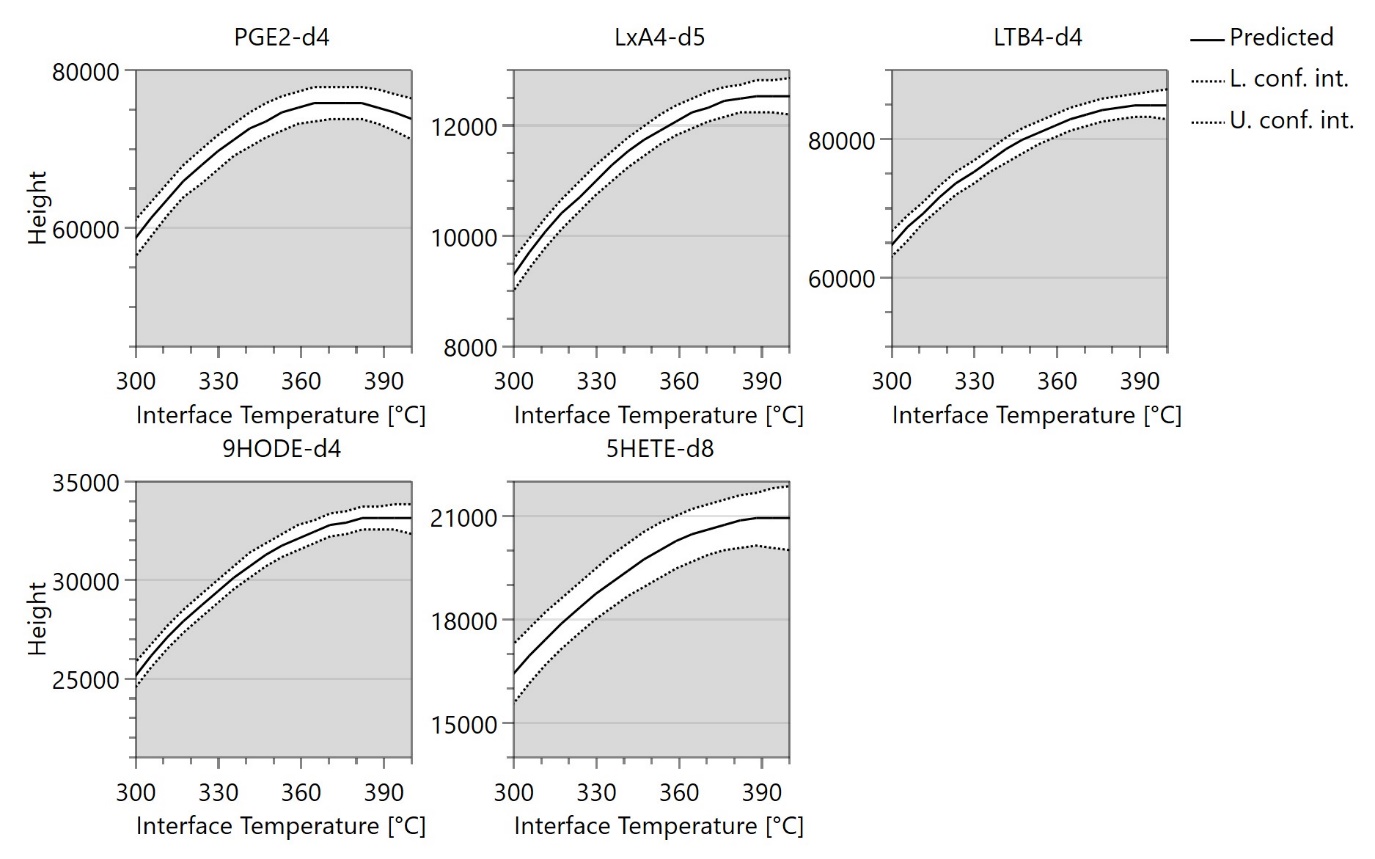


**Figure S3** Main Effect plot for Interface Temperature. All analytes showing a quadratic effect with optimal temperatures between 360 and 400°C. A closer look reveals the different maxima of PGE_2_-d_4_ compared to the other analytes. While PGE_2_-d_4_ does not tolerate higher temperatures, all other analytes, including mono, di and trihydroxylated species, benefit from higher IntT.


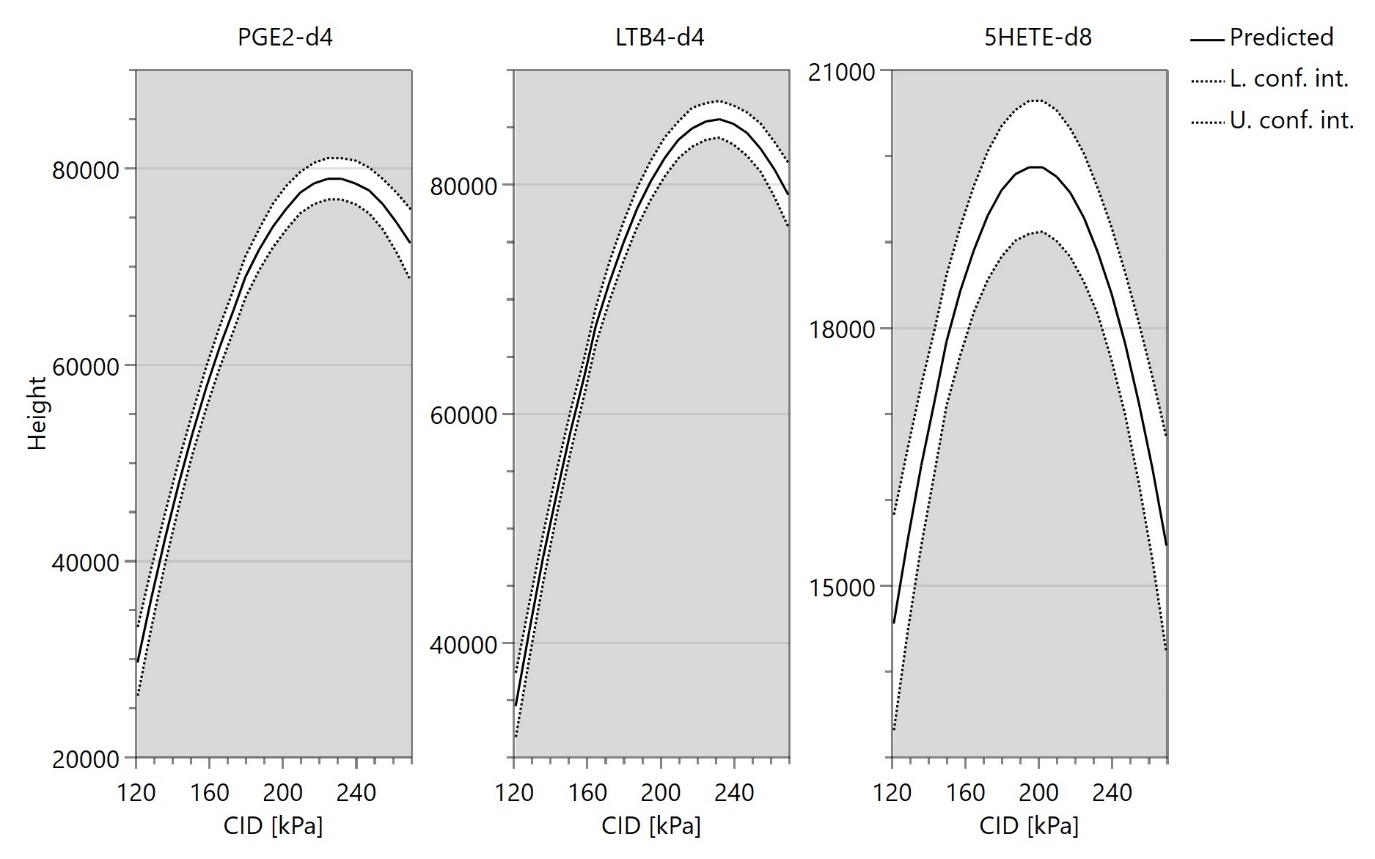


**Figure S4** Main Effect plot for PGE_2_-d_4_, LTB_4_-d_4_ and 5HETE-d_8_ showing the gap between optimal CID setting. LTB_4_-d_4_ and PGE_2_-d_4_ intensity reaches its optimum at around 230 kPa, whereas 5HETE-d_8_ benefits from a lower CID setting at 200 kPa.


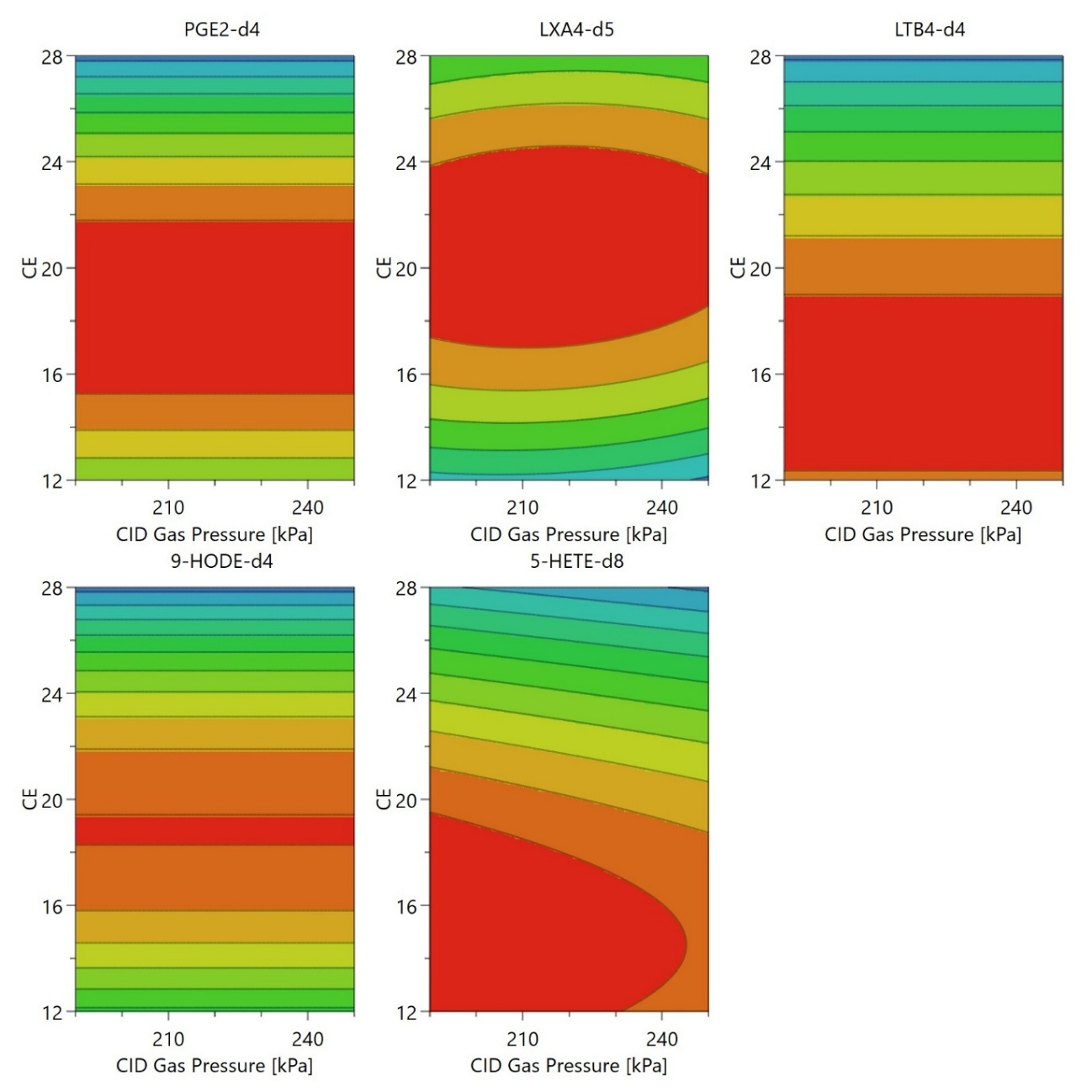

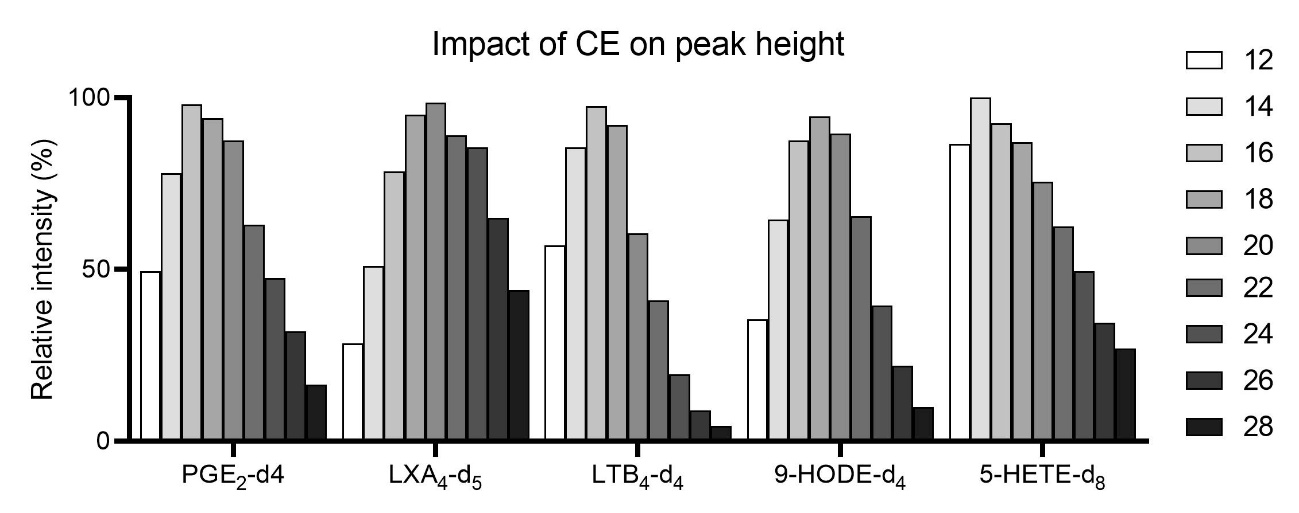


**b**

**a**

**Figure S5** a) Response contour plots of the five tested oxylipins, showing the relationship between CID gas pressure (x-axis) and collision energy (y-axis). The large impact of collision energy outweigh the influence of CID in this experimental setup. b) Optimal CE determination at fixed CID gas pressure of 222 kPa.


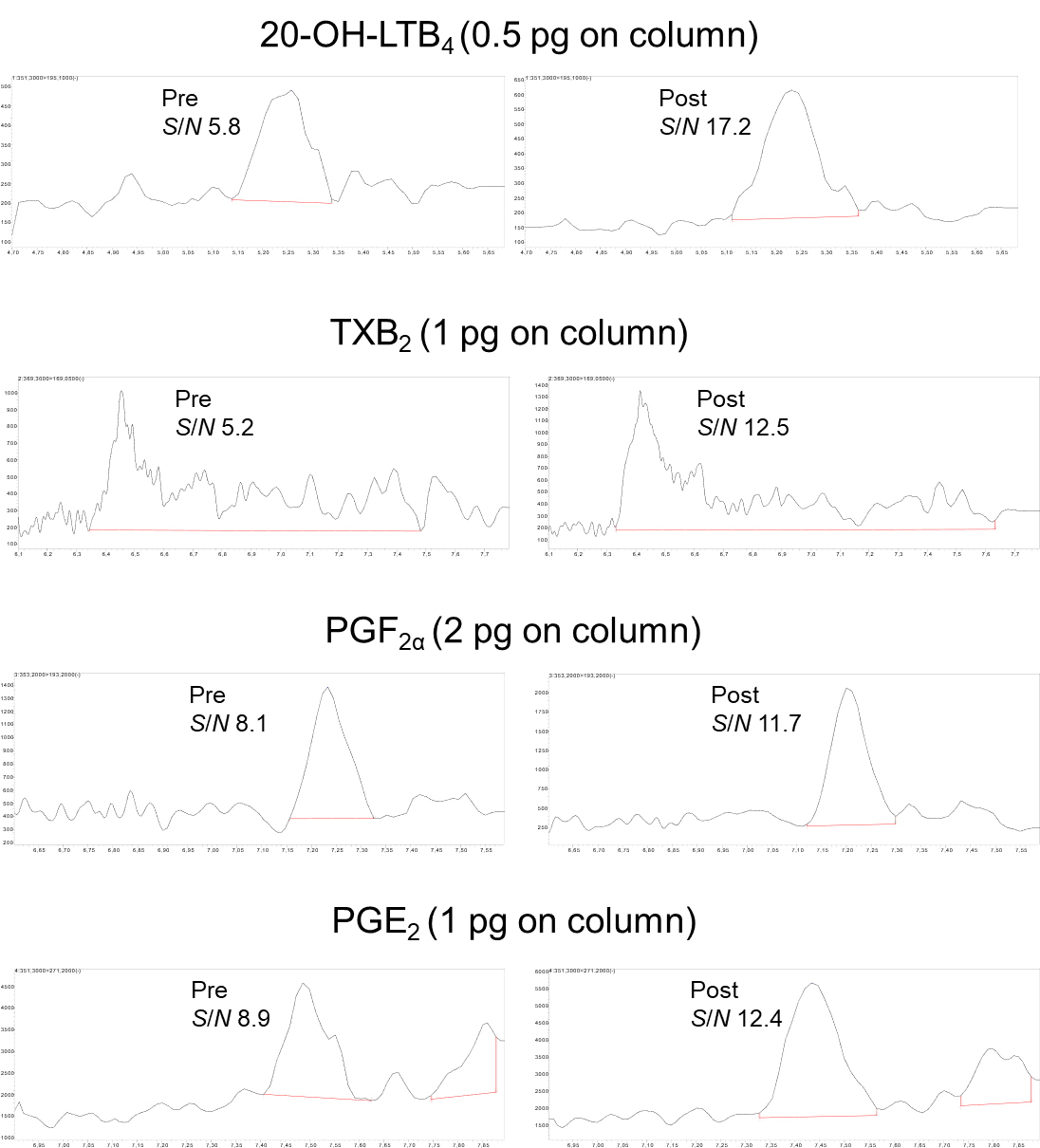


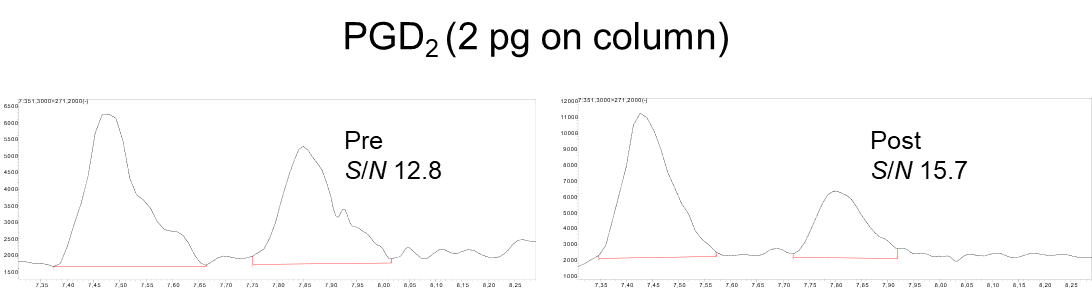


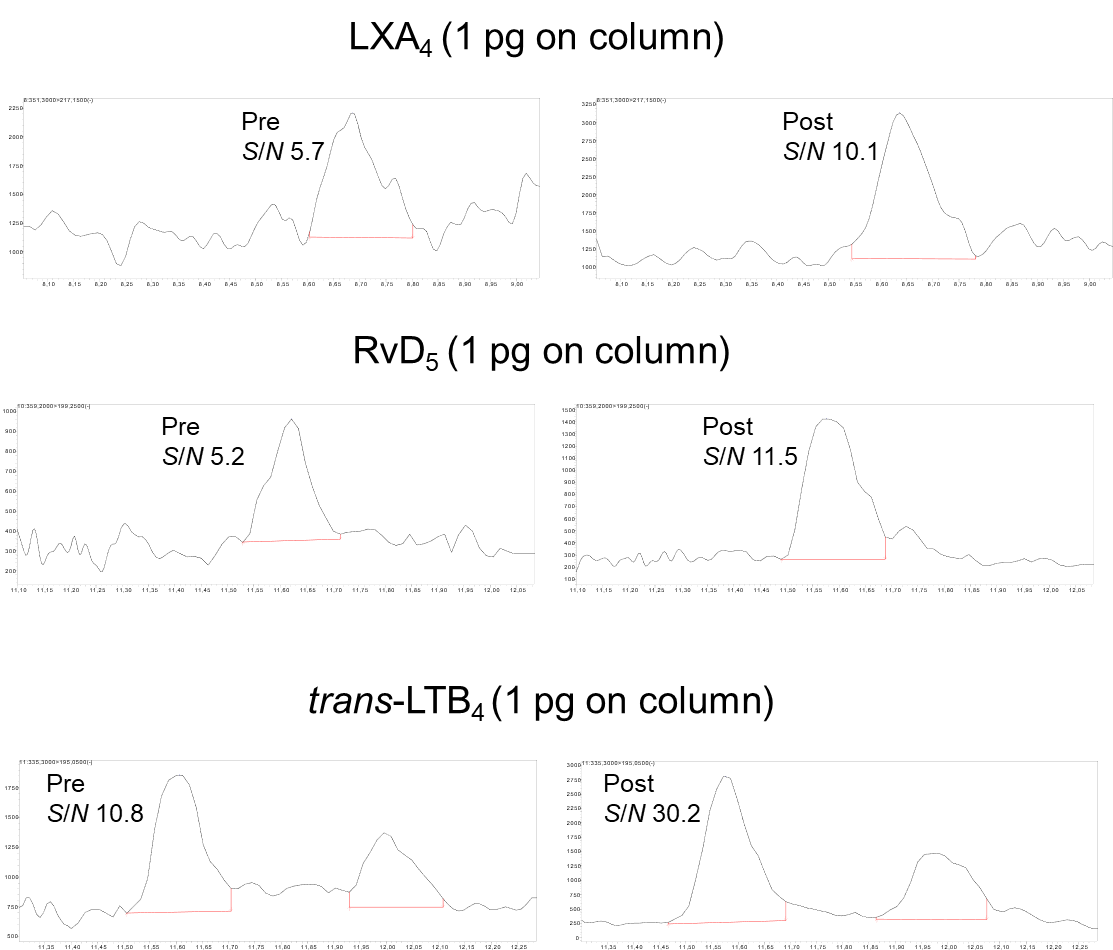


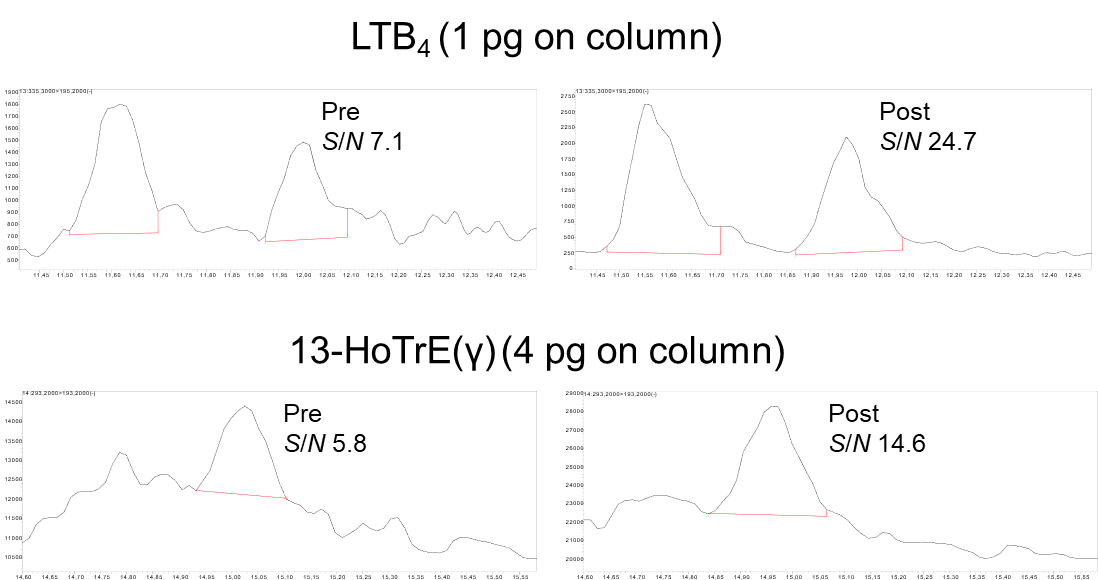


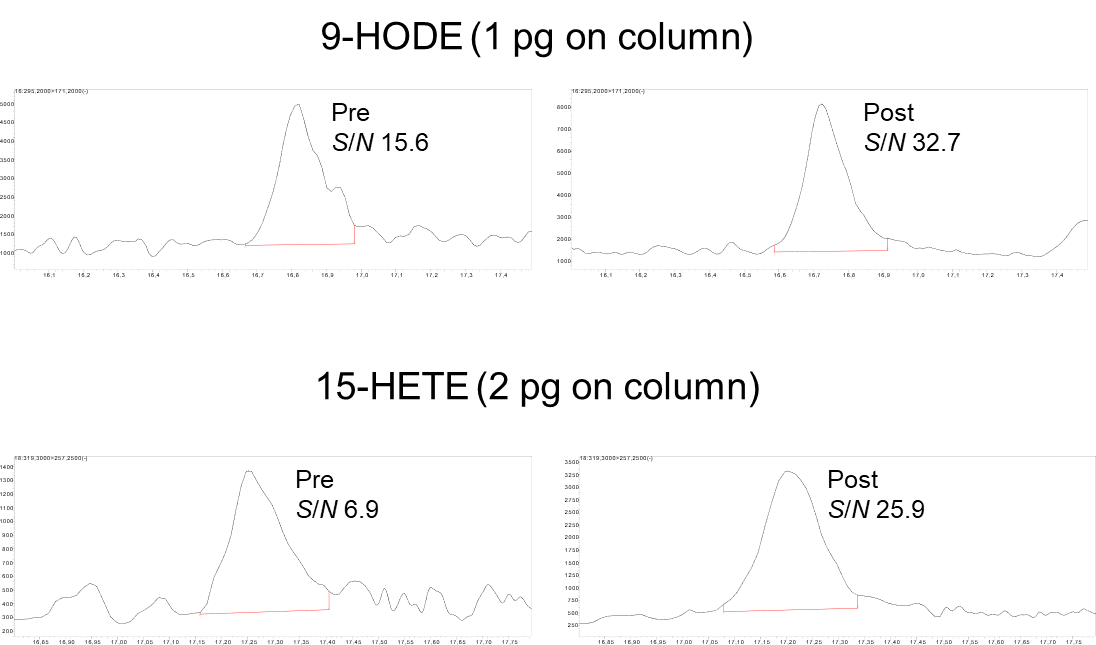


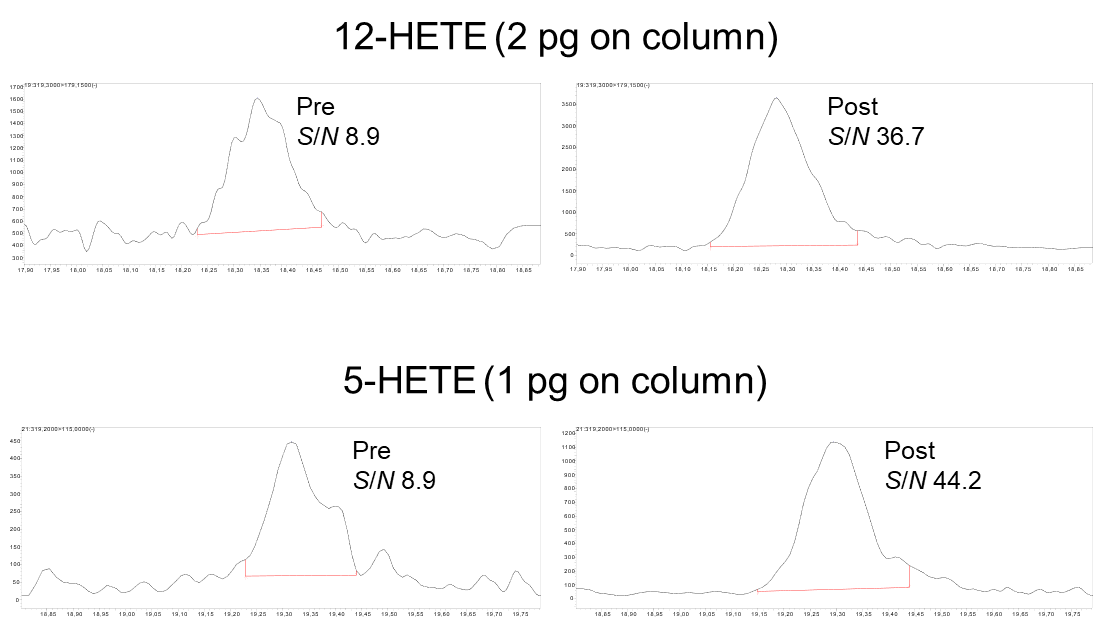


**Figure S6** Comparison of chromatograms obtained with pre (left) and post (right) optimization conditions. DoE optimization of the ESI-MS/MS improved *S*/*N* ratios and facilitates detection of low abundant oxylipins. Dwell time of the instrument was set to acquire 15 to 20 data points/peak. Data shown as raw data (no smoothing applied).

Table S1: Mass transition, MRM parameter, retention time and LOQ for all oxylipins included in this study

| **Analyte** | **Mass transition** | | **MS paramter** | | | **tR** | **LOQ pre optimisation** | | **LOQ post optimisation** | |
| --- | --- | --- | --- | --- | --- | --- | --- | --- | --- | --- |
|  | Q1 | Q3 | Q1 Pre Bias [V] | CE | Q3 Pre Bias [V] | min | pg/ml | pg on column | pg/ml | pg on column |
| 20-OH-LTB_4_ | 351.3 | 195.1 | 30 | 18 | 12 | 5.3 | 48.8 | 0.5 | 48.8 | 0.5 |
| TXB_2_ | 369.3 | 169.0 | 29 | 24 | 12 | 6.5 | 97.7 | 1 | 48.8 | 0.5 |
| PGF_2α_ | 353.2 | 193.2 | 30 | 20 | 13 | 7.3 | 195.3 | 2 | 97.7 | 1 |
| PGE_2_ | 351.3 | 271.2 | 16 | 18 | 18 | 7.5 | 97.7 | 1 | 48.8 | 0.5 |
| PGD_2_ | 351.3 | 271.2 | 16 | 18 | 18 | 7.9 | 97.7 | 1 | 97.7 | 1 |
| LXA_4_ | 351.3 | 217.1 | 28 | 22 | 14 | 8.9 | 97.7 | 1 | 97.7 | 1 |
| RvD_5_ | 359.2 | 199.2 | 14 | 18 | 12 | 11.5 | 97.7 | 1 | 48.8 | 0.5 |
| *t*-LTB_4_ | 335.3 | 195.0 | 15 | 16 | 12 | 11.8 | 97.7 | 1 | 48.8 | 0.5 |
| LTB_4_ | 335.3 | 195.2 | 14 | 17 | 12 | 12.3 | 195.3 | 2 | 48.8 | 0.5 |
| 13-HoTrE_γ_ | 293.2 | 193.2 | 24 | 14 | 11 | 14.9 | 390.6 | 4 | 195.3 | 2 |
| 9-HODE | 295.2 | 171.2 | 12 | 19 | 10 | 16.9 | 97.7 | 1 | 97.7 | 1 |
| 15-HETE | 319.3 | 257.2 | 27 | 15 | 16 | 17.4 | 195.3 | 2 | 97.7 | 1 |
| 12-HETE | 319.3 | 179.1 | 19 | 15 | 11 | 18.5 | 195.3 | 2 | 48.8 | 0.5 |
| 5-HETE | 319.3 | 115.0 | 28 | 15 | 11 | 19.4 | 97.7 | 1 | 24.4 | 0.24 |
| PGE_2_-d_4_ | 355.3 | 275.2 | 16 | 18 | 18 | 7.5 | n.d. | n.d. | n.d. | n.d. |
| LXA_4_-d_5_ | 356.3 | 222.2 | 30 | 22 | 14 | 8.7 | n.d. | n.d. | n.d. | n.d. |
| LTB_4_-d_4_ | 339.3 | 197.2 | 29 | 17 | 13 | 12.1 | n.d. | n.d. | n.d. | n.d. |
| 9HODE-d_4_ | 299.3 | 281.3 | 25 | 19 | 12 | 16.8 | n.d. | n.d. | n.d. | n.d. |
| 5HETE-d_8_ | 327.3 | 116.0 | 27 | 15 | 11 | 19.3 | n.d. | n.d. | n.d. | n.d. |

Table S2: Design matrix for screening design (FFD, Res IV). 16 runs and three center points (*) were evaluated.

| **Exp  No** | **Run Order** | **CID Gas Pressure [kPa]** | **Interface Tempera-ture [°C]** | **Interface Voltage [kV]** | **Desol-vation Line [°C]** | **Heat-block [°C]** | **Heating Gas [L/min]** | **Drying Gas [L/min]** |
| --- | --- | --- | --- | --- | --- | --- | --- | --- |
| 1 | 10 | 140 | 200 | 2 | 200 | 300 | 5 | 5 |
| 2 | 14 | 270 | 200 | 2 | 200 | 500 | 5 | 10 |
| 3 | 8 | 140 | 400 | 2 | 200 | 500 | 10 | 5 |
| 4 | 2 | 270 | 400 | 2 | 200 | 300 | 10 | 10 |
| 5 | 16 | 140 | 200 | 4 | 200 | 500 | 10 | 10 |
| 6 | 1 | 270 | 200 | 4 | 200 | 300 | 10 | 5 |
| 7 | 19 | 140 | 400 | 4 | 200 | 300 | 5 | 10 |
| 8 | 18 | 270 | 400 | 4 | 200 | 500 | 5 | 5 |
| 9 | 9 | 140 | 200 | 2 | 300 | 300 | 10 | 10 |
| 10 | 17 | 270 | 200 | 2 | 300 | 500 | 10 | 5 |
| 11 | 3 | 140 | 400 | 2 | 300 | 500 | 5 | 10 |
| 12 | 7 | 270 | 400 | 2 | 300 | 300 | 5 | 5 |
| 13 | 12 | 140 | 200 | 4 | 300 | 500 | 5 | 5 |
| 14 | 5 | 270 | 200 | 4 | 300 | 300 | 5 | 10 |
| 15 | 11 | 140 | 400 | 4 | 300 | 300 | 10 | 5 |
| 16 | 15 | 270 | 400 | 4 | 300 | 500 | 10 | 10 |
| 17* | 13 | 205 | 300 | 3 | 250 | 400 | 7.5 | 7.5 |
| 18* | 4 | 205 | 300 | 3 | 250 | 400 | 7.5 | 7.5 |
| 19* | 6 | 205 | 300 | 3 | 250 | 400 | 7.5 | 7.5 |

Table S3: Design matrix for optimization design (CCO). 14 runs and three center points (*) were done.

| **Exp No** | **Run Order** | **CID Gas Pressure [kPa]** | **Interface Temperature [°C]** | **Interface Voltage [kV]** |
| --- | --- | --- | --- | --- |
| 1 | 3 | 140 | 300 | 2 |
| 2 | 13 | 250 | 300 | 2 |
| 3 | 1 | 140 | 400 | 2 |
| 4 | 10 | 250 | 400 | 2 |
| 5 | 8 | 140 | 300 | 3 |
| 6 | 14 | 250 | 300 | 3 |
| 7 | 6 | 140 | 400 | 3 |
| 8 | 11 | 250 | 400 | 3 |
| 9 | 17 | 120 | 350 | 2.5 |
| 10 | 9 | 270 | 350 | 2.5 |
| 11 | 7 | 195 | 282 | 2.5 |
| 12 | 2 | 195 | 400 | 2.5 |
| 13 | 4 | 195 | 350 | 1.8 |
| 14 | 12 | 195 | 350 | 3.7 |
| 15* | 16 | 195 | 350 | 2.5 |
| 16* | 15 | 195 | 350 | 2.5 |
| 17* | 5 | 195 | 350 | 2.5 |

Table S4: Instrument setting tested to compare the effect of single parameter change.

| **Setup** | **Interface Voltage** | **Interface Tempe-**  **rature** | **CID gas pres-sure** | **Drying gas** | **Heating gas** | **Heating block** | **Desol-vation line** |
| --- | --- | --- | --- | --- | --- | --- | --- |
|  | **[kV]** | **[°C]** | **[kPa]** | **[L/min]** | **[L/min]** | **[°C]** | **[°C]** |
| Standard | 3 | 300 | 270 | 10 | 10 | 400 | 250 |
| Interface Voltage | 2 | 300 | 270 | 10 | 10 | 400 | 250 |
| Interface Temperature | 3 | 380 | 270 | 10 | 10 | 400 | 250 |
| CID Gas Pressure | 3 | 300 | 222 | 10 | 10 | 400 | 250 |
| Final | 2 | 380 | 222 | 10 | 10 | 400 | 250 |
